# The Roles of Dipeptidyl Peptidase 4 (DPP4) and DPP4 Inhibitors in Different Lung Diseases: New Evidence

**DOI:** 10.3389/fphar.2021.731453

**Published:** 2021-12-09

**Authors:** Tianli Zhang, Xiang Tong, Shijie Zhang, Dongguang Wang, Lian Wang, Qian Wang, Hong Fan

**Affiliations:** Department of Respiratory and Critical Care Medicine, West China Hospital/West China School of Medicine, Sichuan University, Chengdu, China

**Keywords:** dipeptidyl peptidase 4 (DPP4), DPP4 inhibitors, infectious lung disease, non-infectious disease, COVID-19, pulmonary fibrosis

## Abstract

CD26/Dipeptidyl peptidase 4 (DPP4) is a type II transmembrane glycoprotein that is widely expressed in various organs and cells. It can also exist in body fluids in a soluble form. DPP4 participates in various physiological and pathological processes by regulating energy metabolism, inflammation, and immune function. DPP4 inhibitors have been approved by the Food and Drug Administration (FDA) for the treatment of type 2 diabetes mellitus. More evidence has shown the role of DPP4 in the pathogenesis of lung diseases, since it is highly expressed in the lung parenchyma and the surface of the epithelium, vascular endothelium, and fibroblasts of human bronchi. It is a potential biomarker and therapeutic target for various lung diseases. During the coronavirus disease-19 (COVID-19) global pandemic, DPP4 was found to be an important marker that may play a significant role in disease progression. Some clinical trials on DPP4 inhibitors in COVID-19 are ongoing. DPP4 also affects other infectious respiratory diseases such as Middle East respiratory syndrome and non-infectious lung diseases such as pulmonary fibrosis, lung cancer, chronic obstructive pulmonary disease (COPD), and asthma. This review aims to summarize the roles of DPP4 and its inhibitors in infectious lung diseases and non-infectious diseases to provide new insights for clinical physicians.

## Introduction

Dipeptidyl peptidase 4 (DPP4), also called CD26, is a serine protease that is widely distributed in various organs and cells. DPP4 can also be found in the plasma and body fluids in a soluble form (Shao et al., 2020). It participates in various physiological and pathological processes of the body, such as inflammation, energy metabolism, immune regulation, cell adhesion, and apoptosis (Klemann et al., 2016). DPP4 inhibitor, as a hypoglycemic agent, was approved by the Food and Drug Administration (FDA) for the treatment of type 2 diabetes mellitus (T2DM). Recent studies have found more biological functions of DPP4 beyond glucose control (Hildebrandt et al., 2001; Jae-Hwi; Ohnuma et al., 2011; Schuetz et al., 2015; Jang et al., 2017). DPP4 is frequently expressed on the surface of the epithelium, vascular endothelial cells, and alveolar macrophages in the lung (Meyerholz et al., 2016). In addition, more evidence has shown the underlying functions of DPP4 and its inhibitors in infectious lung diseases and non-infectious lung diseases (Meyerholz et al., 2016; Shiobara et al., 2016; Suzuki et al., 2017; Bishnoi et al., 2019). Here, we summarized the emerging roles of DPP4 and DPP4 inhibitors in lung diseases to provide new insights into clinical work.

## Literature Search

We performed a literature search in PubMed database up to October 23, 2021. The key search terms were as follows: “DPP4 or DPPIV or CD26”, “DPP4 inhibitor or Vildagliptin or Saxagliptin or Sitagliptin or Alogliptin,” “respiratory disease or lung disease,” “lung cancer,” “pulmonary fibrosis,” “pulmonary hypertension,” “asthma,” “chronic obstructive pulmonary disease or COPD,” “SARS-CoV2 or COVID-19,” “Middle East Respiratory Syndrome or MERS”. After an initial screening of the title and abstract, the full text of all the relevant articles in English language about the roles of DPP4 and DPP4 inhibitors in lung diseases were included. Additionally, we also included some relevant articles from references to ensure a comprehensive search. Finally we reviewed the available literature and presented it.

## DPP4 Molecule and Expression

**FIGURE 1:**
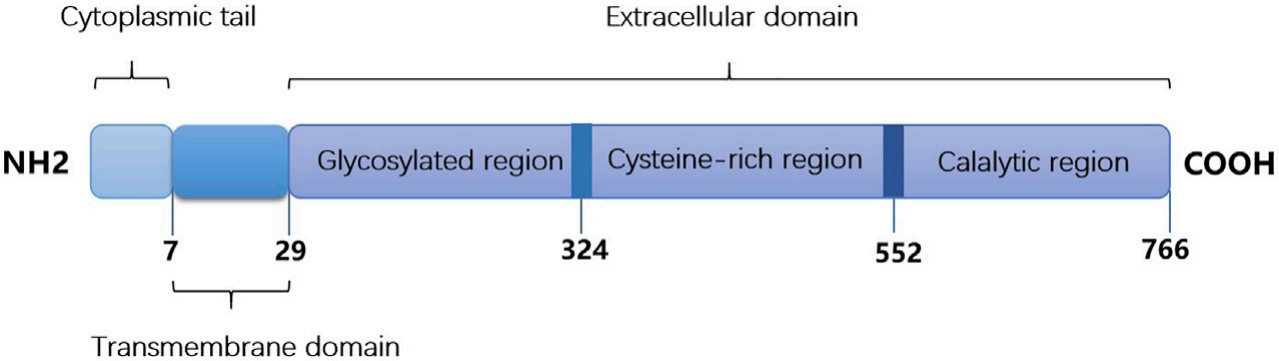
The main structure of DPP4/CD26. It consists of cytoplasmic tail (1-6 amino acids), transmembrane domain (7–29 amino acids) and extracellular domain (30–766 amino acids).

As a member of the subfamily S9B of serine peptidases, DPP4/CD26 is a highly conserved type II transmembrane glycoprotein, which consists of a 6-residue N–terminal cytoplasmic tail, a 22 amino acid transmembrane, and extracellular domain (Matteucci and Giampietro, 2009). Figure 1 shows the main structure of DPP4/CD26. As a hydrophobic domain and a part of the putative signal peptide, the transmembrane domain is not cleaved during synthesis. The extracellular domain cleaves dipeptide after the second position from the N-terminus of peptides with proline or alanine, which indicates dipeptidyl peptidase activity (Fleischer, 1994). Soluble DPP4 can also be found in body fluids due to the removal of intracellular and transmembrane regions, and it still has enzymatic activity (Klemann et al., 2016). Many peptides, such as cytokines, chemokines, and neuropeptides, can be cut by membrane or soluble DPP4, which further regulates their biological functions (Anderluh et al., 2016; Beckers et al., 2017).

DPP4 is extensively expressed in many tissues, such as the lung, intestine, kidney, liver, brain, thymus, bladder, pancreas, spleen, uterus, lymph node, epithelium, endothelium, and immune cells (Gorrell, 2005). DPP4 was demonstrated to be broadly distributed in the lung parenchyma and the surface of the epithelium, vascular endothelium, and fibroblasts of human bronchi, indicating that DPP4 may play a significant role in regulating physiological and pathological functions in the lung (Van Der Velden and Hulsmann, 1999). DPP4 has been reported to be expressed in immune cells such as T cells, B cells, natural killer (NK) cells, and macrophages (Nieto-Fontarigo et al., 2019). CD4^+^ T cells express DPP4 more frequently than CD8^+^ T cells, which might be related to the T helper type 1 (Th1)-like phenotype (Willheim et al., 1997). DPP4 was detected to be low in NK cells, while it increased after cytokine stimulation (Yamabe et al., 1997). Likewise, DPP4 can be detected in approximately 50% of B cells after activation (Bühling et al., 1995). Therefore, more effects of DPP4 in the immune system have been observed.

## DPP4 Functions

DPP4 plays an important role in regulating body metabolism since it cleaves and inactivates peptides, such as glucagon-like peptide-1 (GLP-1), incretin hormones, and glucose-dependent insulinotropic polypeptide (GIP). And DPP4 inhibitors have been widely used to treat T2DM. In addition, circulating DPP4 concentration was demonstrated to be higher in individuals with obesity than in those with normal body weight (Rohmann et al., 2021). Barchetta et al. found higher circulating DPP4 activity in non-alcoholic fatty liver disease (NAFLD) patients than in non-NAFLD patients (Barchetta et al., 2021). Sitagliptin, a DPP4 inhibitor, was reported to decrease plasma apolipoprotein B and triglyceride levels in patients with T2DM, indicating the function of DPP4 in regulating lipid metabolism (Tremblay et al., 2011).

DPP4 also participates in inflammation, as it can cleave chemokines and cytokines (Klemann et al., 2016). Wronkowitz et al. observed that sDPP4 could increase the secretion of pro-inflammatory cytokines, such as interleukin (IL)-6, IL-8, and MCP-1, and that inflammation could be prevented by inhibiting DPP4 *in vitro* (Wronkowitz et al., 2014). sDPP4 might stimulate inflammation through the upregulation of toll-like receptors (D. S. Lee et al., 2016). Sitagliptin has been demonstrated to decrease serum DPP4 activity and reduce the production of pro-inflammatory cytokines in LPS-induced lung injury in mice (Kawasaki et al., 2018).

DPP4 is a surface marker expressed in senescent cells (Kim et al., 2017). Chen et al. reported increased DPP4 levels in senescent endothelial cells and aging arteries, and DPP4 inhibition could decrease the production of senescent markers such as p21 and p53 (Z. Chen et al., 2020b). In addition, the binding of DPP4 and adenosine deaminase (ADA) can activate the DPP4/ADA pathway to modulate T cell functions (Hatano et al., 2013). DPP4 has also been demonstrated to be related to CD8^+^ T cell co-stimulation, B cell activation, macrophage polarization, and NK cell proliferation, playing an emerging role in modulating immune functions (Shingu et al., 2003; Yan et al., 2003; Hatano et al., 2013; Zhuge et al., 2016). Regarding its role in the immune system, DPP4 was shown to participate in the pathogenesis of autoimmune diseases (rheumatoid arthritis, inflammatory bowel disease) and graft-versus-host disease (GVHD) (Pan et al., 2021).

## DPP4 in Infectious Lung Diseases

### SARS-Cov-2 Infection

COVID-19 is caused by SARS-Cov-2 and has been a pandemic disease threatening the public health of the global population since December 2019. Naveen et al. predicted the molecular model of SARS-Cov-2 and found that the S1 domain of SARS-Cov-2 spike glycoprotein might interact with DPP4 of host cells directly, which implies the effects of DPP4 in COVID-19 (Vankadari and Wilce, 2020). Tai et al. identified the receptor-binding domain (RBD) in the S1 region of SARS-Cov-2, which can bind to angiotensin-converting enzyme 2 (ACE2) in humans or bats. However, the RBD of the S1 domain did not exhibit binding activity to DPP4 in human cells (Tai et al., 2020). Although there is a lack of evidence for the combination of the S1 protein with DPP4, the roles of DPP4 and DPP4 inhibitors in COVID-19 have been increasingly realized. Diabetes is a common comorbidity of COVID-19 and is a risk factor for poor prognosis (F. Zhou et al., 2020a). Higher levels of DPP4 might be correlated with the occurrence and severity of COVID-19 (Radzikowska et al., 2020). DPP4 inhibition may decrease lung damage by reducing the production of cytokines, exhibiting anti-inflammatory effects and regulating glucose metabolism (Solerte et al., 2020a). Acute respiratory distress syndrome (ARDS) is the leading cause of death in COVID-19 patients, and DPP4 inhibitors may be a new treatment approach because they can decrease the production of inflammatory factors such as IL-1β, TNF-α, and IL-6 (Kawasaki et al., 2018). A retrospective study including 338 patients showed that sitagliptin, a DPP4 inhibitor, can reduce mortality and improve clinical outcomes in COVID-19 patients with T2DM. In addition, plasma C-reactive protein (CRP) and procalcitonin levels were also reduced in sitagliptin-treated patients compared to those in the control group, which indicated its anti-inflammatory and immunomodulatory effects (Solerte et al., 2020b). However, a multicenter retrospective analysis in China reported that there was no significant decrease in the occurrence of poor outcomes when using DPP4 inhibitors in COVID-19 patients with T2DM compared to non-users. Moreover, they did not observe significant differences in cytokine concentrations like TNF-α and IL-6 between the two groups (J. H. Zhou JH. et al., 2020). Strollo drew a similar conclusion after analyzing data from the Italian National Institute of Health (Strollo et al., 2021). The difference in the occurrence of mechanical ventilation or death was found to be insignificant between the DPP4 inhibitor and non-DPP4 inhibitor treatment groups (27.7% versus 28.6%, respectively) (Roussel et al., 2021). Hariyanto performed a meta-analysis on the application of DPP4 inhibitors in COVID-19 patients with T2DM, and the results showed that there was no beneficial outcome compared to patients without DPP4 inhibitor treatment (Hariyanto and Kurniawan, 2021). The results of a meta-analysis conducted by Pal and Rakhmat both demonstrated that DPP4 inhibitors could reduce mortality and improve the outcome of these patients (Pal et al., 2021; Rakhmat et al., 2021). However, the majority were retrospective, observational studies, and the confounding factors were not well balanced, including inclusion criteria and baseline characteristics such as comorbidity and inflammation levels. However, these results are controversial. Thus, large-scale, randomized controlled clinical trials are needed, and some clinical trials are ongoing or recruiting. Table 1 summarized the results of the clinical effects of DPP4 inhibitors in COVID-19 patients with T2DM in recent studies.

**TABLE 1:** Clinical outcomes of DPP4 inhibitors in COVID-19 patients with T2DM.

Reference	Research type	Country	Sample size (users/non-users)	Intensive care or mortality (users/non-users)
Solerte et al. (2020a)	Retrospective	Italy	338 (169/169)	18%/37%
J. H. Zhou et al., 2020b	Retrospective	China	444 (111/333)	1.8%/3.3%
Rhee et al. (2021)	HIRA database	Korea	832 (263/569)	3.4%/4.4%
	NHIS database	Korea	704 (175/529)	8%/11.5%
Y. Chen et al., 2020a	Retrospective	China	120 (20/100)	25%/14%
Mirani et al. (2020)	Retrospective	Italy	90 (11/79)	9.1%/46.8%
Wargny et al. (2021)	Retrospective	France	2794 (615/2179)	18.3%/21.3%
Fadini et al. (2020)	Retrospective	Italy	85 (9/76)	11.1%/13.9%
Noh et al. (2021)	Cohort	—	586 (453/133)	10.4%/16.5%
Pérez-Belmonte et al. (2020)	Retrospective	Spain	1589 (180/1409)	41.7%/31.2%
Roussel et al. (2021)	Cohort	France	2449 (596/1853)	9.7%/11.7%
Silverii et al. (2021)	Retrospective	Italy	159 (13/146)	38.5%/37.0%
Israelsen et al. (2021)	Retrospective	Denmark	928 (284/644)	10.6%/3.3%
Cariou et al. (2020)	Retrospective	France	1317 (285/1032)	9.5%/10.9%

HIRA, health insurance review and assessment service; NHIS: national health insurance service.

### MERS

Middle East respiratory syndrome (MERS) was first reported in Saudi Arabia in 2012 (Zaki et al., 2012). As a receptor, membrane DPP4 can bind to the S1 domain in MERS-CoV and mediate virus entry into host cells (N. Wang et al., 2013). As the DPP4 enzymatic position is different from the binding site (blades 4 and 5), DPP4 inhibitors such as vildagliptin and sitagliptin were ineffective in preventing MERS-CoV infection (Raj et al., 2013). Anti-DPP4 antibodies targeting the binding site of DPP4, such as YS110, blocked the entry of MERS-CoV (Ohnuma et al., 2013). Adenosine deaminase (ADA) can competitively bind to DPP4 to prevent the combination of DPP4 with the S1 domain of MERS-CoV (Raj et al., 2014). Therefore, ADA has become a potential agent to block viral infection. In addition, the role of soluble DPP4 (sDPP4) was demonstrated. Contrary to the primary expectation, sDPP4 levels were found to be lower in MERS patients than in healthy individuals, which indicates that exogenous sDPP4 may be a new approach to treat MERS (Inn et al., 2018). Abdullah confirmed that increased levels of sDPP4 in serum indicated higher resistance to MERS-CoV infection *in vitro*, and recombinant soluble human DPP4 (shDPP4) proteins can reduce the infection in mice (Algaissi et al., 2019). Recombinant shDPP4 might be a promising agent for MERS therapy, and more evidence is needed.

## DPP4 IN NON-INFECTIOUS Lung Diseases

### Pulmonary Fibrosis

Idiopathic pulmonary fibrosis (IPF) is a chronic progressive respiratory disease characterized by respiratory failure at the final stage. Irreversible lung injury and limited treatment options have caused high mortality, with a survival rate of only 20% over 5 years (Richeldi et al., 2017). DPP4 was found to be a marker of activated fibroblasts, and DPP4 inhibition attenuated kidney fibrosis, dermal fibrosis, and liver fibrosis (Gangadharan Komala et al., 2016; Lay et al., 2019; S. Y.; Lee et al., 2020; Soare et al., 2020). Yang found that the DPP4 inhibitor vildagliptin can ameliorate pulmonary fibrosis by inhibiting the production of extracellular matrix (ECM) and inflammatory cells in mice (Liu and Qi, 2020). Another experiment performed by Suzuki et al. showed that DPP4 was increased in pulmonary vascular endothelial cells (PVECs) in pulmonary fibrosis models. Vildagliptin exhibited anti-fibrotic effects by inhibiting endothelial-to-mesenchymal transition (EndMT) *in vivo* and *in vitro* (Suzuki et al., 2017). In addition, epithelial and endothelial senescence has been reported to participate in pulmonary fibrosis, suggesting that anti-aging drugs may be effective (Schafer et al., 2017; Yao et al., 2021). DPP4 inhibition can also alleviate cellular senescence because it is a marker of senescent cells (Z. Chen et al., 2020b). However, whether DPP4 inhibition can ameliorate pulmonary fibrosis via an anti-senescence mechanism is unknown. And it is still unclear about the effects of DPP4 inhibition in patients with pulmonary fibrosis.

### Pulmonary Hypertension

Pulmonary hypertension is defined as elevated pulmonary arterial pressure >25 mmHg, which affects approximately 1% of people worldwide, especially the elderly (Hoeper et al., 2016). Pulmonary hypertension is characterized by abnormal proliferation of endothelial cells and smooth muscle cells (SMCs), which eventually develops into right ventricular failure (Anderluh et al., 2019). DPP4 was found to be highly expressed in pulmonary arterial SMCs, and the Akt/mTORC1 and NF-κB pathways were demonstrated to mediate the development of pulmonary arterial SMC and hypoxia-induced pulmonary hypertension (Li et al., 2019). Xu et al. found that the DPP4 inhibitor sitagliptin decreased the infiltration of inflammatory cells and EndMT and ameliorated pulmonary arterial remodeling in rats, in which the PTEN-Akt-MAPK signaling pathway may play a role in progression (Xu et al., 2018). In addition, a GLP-1 receptor antagonist was reported to neutralize the effects of DPP4 inhibition in pulmonary hypertension, which indicated that GLP-1 participated in the protective effects of DPP4 inhibitors to improve pulmonary arterial remodeling (J. Wang et al., 2019). Pirozzi et al. reported the effects of the DPP4 inhibitor vildagliptin in treating a 74-year-old female patient with T2DM and pulmonary hypertension. They observed an improvement in pulmonary symptoms and a reduction in systolic pressure of the right ventricle (SPRV) after 6 months (Fontes Pirozzi et al., 2017). DPP4 inhibitor probably exerted its role in pulmonary hypertension by promoting vasodilatation of the pulmonary artery and producing anti-inflammatory effects. Thus, DPP4 inhibition might be a potential therapeutic target for pulmonary hypertension, and clinical trials are needed.

### Asthma

Bronchial asthma is characterized by chronic airway inflammation, with eosinophils, mast cells, neutrophils, and T lymphocyte infiltration. DPP4 levels are elevated in airway epithelial cells in asthma patients, and DPP4 can stimulate the proliferation of lung fibroblasts and bronchial SMCs *in vitro* (Shiobara et al., 2016). Kruschinski found that DPP4-dependent T cells were recruited to the lung in a rat asthma model, while in DPP4-deficient rats T cells obviously decreased (Kruschinski et al., 2005). This indicates that DPP4 may participate in the occurrence of asthma by regulating T cells. Interleukin-13 (IL-13), secreted by Th2 cells, has been shown to be related to airway inflammation and allergy in asthma (Mitchell et al., 2010). A whole-transcriptome RNA sequencing study of nasal epithelial cells in children with asthma indicated that the DPP4 gene had a positive relationship with IL-13 mRNA level (Poole et al., 2014). In addition, DPP4 mRNA was shown to be induced by IL-13, indicating the potential role of DPP4 in asthma (Ranade et al., 2016). The DPP4 inhibitor sitagliptin can ameliorate airway remodeling by decreasing the production of IL-13 and the number of inflammatory cells and fibrotic-related factors such as TGF-β and NF-κB in mice with chronic asthma (Nader, 2015). Another DPP4 inhibitor, saxagliptin, was reported to alleviate airway inflammation in ovalbumin (OVA)-induced asthma in mice via the NF-κB and TLR4 pathways (Helal et al., 2019). However, a retrospective study conducted by Colice showed that there was no significant difference in the asthma control rate between DPP4 inhibitor users and non-users in asthma patients with T2DM. This might be related to the non-specific distribution of oral DPP4 inhibitor (Colice et al., 2017).

### COPD

Chronic obstructive pulmonary disease (COPD) is a common respiratory disease that usually occurs in the elderly and in smokers. Inflammation of the airway and disordered immune functions facilitate the pathogenesis of COPD (Barnes et al., 2003). sDPP4 levels were demonstrated to be lower in COPD patients than in non-COPD patients, and it was expected to be a biomarker for the diagnosis of COPD (Somborac-Bačura et al., 2012; Chang et al., 2016). In contrast, Seys and others found DPP4 mRNA and protein levels were higher in smokers and COPD patients, and DPP4 increase was mainly located in alveolar epithelial cells rather than bronchial and bronchiolar epithelium. In addition, DPP4 level was also related to the stage of COPD and smoking status (Seys et al., 2018). The differences in DPP4 expression in the serum and lungs were unclear. The GLP-1 receptor agonist was found to be effective in decreasing the mortality of COPD female mice, suggesting that the GLP-1 receptor agonist is a promising agent for COPD therapy (Viby et al., 2013). Therefore, DPP4 inhibition may prevent the degradation of GLP-1 by DPP4 and play a role in COPD. However, further experiments and clinical trials should be performed.

### Lung Cancer

Several studies have examined DPP4 expression in various malignancies to determine the role of DPP4 in tumor progression. However, the expression of DPP4 and its functions in tumors differ according to tumor type (Havre et al., 2008). The expression of DPP4 in colorectal cancer, malignant mesothelioma, and hematological malignancies is high and is related to tumor progression (Sato et al., 2005; Pang et al., 2010; Okamoto et al., 2014). DPP4 has been shown to be a tumor suppressor in other cancers, such as melanoma and ovarian cancer (Wesley et al., 1999; Kajiyama et al., 2002). The differences in DPP4 effects might be attributed to the tumor microenvironment and the complex functions of the molecule (Pro and Dang, 2004). Accordingly, as a malignant tumor with high morbidity and mortality, lung cancer is classified into small cell lung cancer (SCLC) and non-small cell lung cancer (NSCLC). DPP4 expression and its role in lung cancer remains controversial. sDPP4 levels are lower in lung cancer tissues than in normal tissues (Sedo et al., 1991). The mRNA and protein expression levels of DPP4 were both decreased in human NSCLC cells compared to normal lung epithelial cells (Wesley et al., 2004). Dimitrova also found that DPP4 activity was lower in human adenocarcinoma cell and squamous cell carcinoma cells than in normal fetal lung-derived P cells (Dimitrova et al., 2012). However, DPP4 expression varies according to the different histologic subtypes of lung cancer. Another study reported that DPP4 activity was high in human lung cancer tissue as well as in human and mouse lung adenocarcinoma cell lines. In addition, vildagliptin can exhibit anti-tumor effects, possibly via regulating macrophage and NK cell activity (J. H. Jang et al., 2019). DPP4 inhibition has been reported to improve progression-free survival (PFS) in advanced airway and colorectal cancer patients with T2DM (Ali et al., 2019). A national database study performed by Bishnoi et al. found that the combined use of DPP4 inhibitors and metformin can significantly increase the survival of lung cancer patients (Bishnoi et al., 2019). The DPP4 inhibitor vildagliptin was also shown to suppress the pulmonary metastasis of colorectal cancer in mice (J. H. Jang et al., 2015). Table 2 listed DPP4 expressions in different lung cancer tissues or cells.

**TABLE 2:** DPP4 expressions in lung cancer tissue or cells.

References	Location	sDPP4 level or activity	mDPP4 level or activity
Sedo et al. (1991)	Human lung cancer tissue	Low	—
Wesley et al. (2004)	Human NSCLC cell lines (H28, H226, H441)	—	Low
Dimitrova et al. (2012)	Human lung adenocarcinoma cell (A549)	—	Low
	Human lung squamous cell carcinoma cell (SK-MES-1)	—	Low
Jang et al. (2019)	Human lung cancer tissue	—	High
	Human lung adenocarcinoma cell (H460)	—	High
	Mouse lung adenocarcinoma cell (LLC)	—	High

The association between DPP4 and malignancy is still debated, and the underlying mechanisms are complex. On the one hand, DPP4 may act as a tumor suppressor in malignant diseases. A study showed that decreased expression of DPP4 in NSCLC cells indicated its tumor suppressor effect, which might be independent of its enzymatic activity (Wesley et al., 2004). Increased DPP4 expression mediates high CD44 and FAP-α expression (Wesley et al., 2004), which could exert suppressive effects on tumor growth and metastasis (Gao et al., 1997; Herrlich et al., 2000). Beckenkamp considered that DPP4 can inhibit tumor metastasis through its enzymatic activity via cleaving and inactivating stromal cell derived factor-1 (SDF-1) (Beckenkamp et al., 2016). In contrast, DPP4 may participate in the carcinogenesis of malignancy. It has been shown that DPP4 participates in tumor growth and invasion and is dependent on its interactive functions with other key molecules and its enzymatic effects. The combination of DPP4 with plasminogen 2-epsilon could promote the secretion of matrix metalloproteinases (MMPs), which are involved in tumor invasion (Gonzalez-Gronow et al., 2001). DPP4 can also exert its tumor progressive effect through induction of epithelial-mesenchymal transition (EMT) and upregulation of EMT markers such as N-cadherin, Slug, Twist, and vimentin (Beckenkamp et al., 2016). Thus, DPP4 function is altered according to specific location, histologic type of tumor, tumor microenvironment, and cofactors. Because of their controversial effects, further studies are needed to clarify the possible roles of DPP4 and DPP4 inhibitors in lung cancer.

## Conclusion and Prospectives

DPP4 is widely expressed in the membranes of the epithelium, endothelium, and fibroblasts of human bronchi. DPP4 and its inhibitors have been shown to play pivotal roles in lung diseases, such as COVID-19 and pulmonary fibrosis. Therefore, targeting DPP4 may provide a novel approach for treating lung diseases in the future.

However, localizing and targeting DPP4 remains a challenge because of its wide distribution, pleiotropic effects, and interaction with other molecules. DPP4 inhibitors can exert systemic effects in the body, and adverse effects are unavoidable. In addition, targeting the enzymatic activity using DPP4 inhibitors or targeting DPP4 protein using anti-DPP4 antibody still requires further study. For instance, DPP4 plays a complicated role in lung cancer of different histologic types via interplay with other key molecules and immune regulation. Thus, it is essential to explore the interaction of DPP4 with the tumor microenvironment, which may provide a new DPP4-targeted therapy strategy for lung cancer patients. Anti-DPP4 antibodies are a new future research topic. As previously mentioned, anti-DPP4 antibodies targeting the binding site of DPP4, such as YS110, blocked the entry of MERS-CoV (Ohnuma et al., 2013). The targeting of the DPP4 protein itself may be valuable for the development of vaccines for MERS and other viral infections. Therefore, further experimental and clinical studies are needed to explore the role of DPP4 in lung diseases to identify novel treatment approaches.
